# Supplementary material for: The interactome of intact mitochondria by cross-linking mass spectrometry provides evidence for coexisting respiratory supercomplexes
Source: Mol Cell Proteomics. 2017 Dec 8;17(2):216–32. doi: 10.1074/mcp.RA117.000470 (PMC5795388; doi:10.1074/mcp.RA117.000470)
Supplement: Supplemental Data [file supp_RA117.000470_133922_0_supp_23978_fzffwf.zip › spectra_annotation/mito_spectra_annotation/273-1-5-1-5-1.pdf]

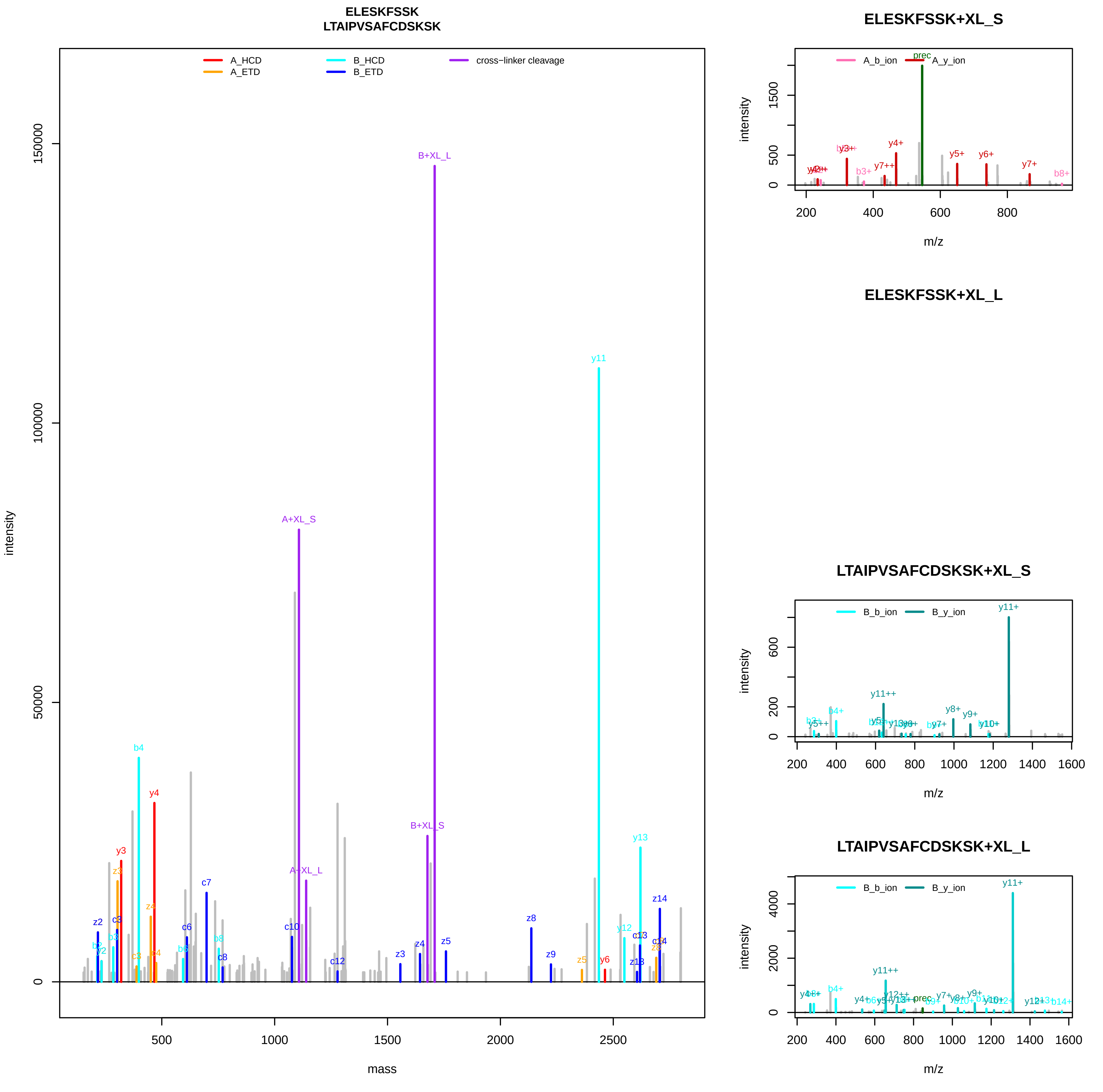

ELESKFSSK
LTAIPVSAFCDSKSK
A_HCD
A_ETD
B_HCD
B_ETD
cross−linker cleavage
intensity
mass
B+XL_L
A+XL_S
B+XL_S
A+XL_L
y11
y13
y12
b4
y4
y3
z3
z4
c7
c6
c3
z2
c10
c12
z3
z4
z5
z8
z9
z5
y6
c13
z13
c14
z14
ELESKFSSK+XL_S
A_b_ion
A_y_ion
prec
y4+
y5+
y6+
y7+
y7++
b3+
b8+
m/z
ELESKFSSK+XL_L
LTAIPVSAFCDSKSK+XL_S
B_b_ion
B_y_ion
y11+
y11++
y8+
y9+
y10+
b4+
LTAIPVSAFCDSKSK+XL_L
y12+
b14+
